# Activation of Neurogenesis in Multipotent Stem Cells Cultured *In Vitro* and in the Spinal Cord Tissue After Severe Injury by Inhibition of Glycogen Synthase Kinase-3

**DOI:** 10.1007/s13311-020-00928-0

**Published:** 2020-09-30

**Authors:** Francisco Javier Rodriguez-Jimenez, Angel Vilches, Maria Amparo Perez-Arago, Eleonora Clemente, Raquel Roman, Juliette Leal, Ana Artero Castro, Santos Fustero, Victoria Moreno-Manzano, Pavla Jendelova, Miodrag Stojkovic, Slaven Erceg

**Affiliations:** 1 Stem Cell Therapies in Neurodegenerative Diseases Lab, Research Center “Principe Felipe”, C/ Eduardo Primo Yufera 3, Valencia, Spain; 2 National Stem Cell Bank-Valencia Node, Biomolecular Resources Platform PRB3, ISCIII, Research Center “Principe Felipe”, C/ Eduardo Primo Yúfera 3, 46012 Valencia, Spain; 3 Organic Molecules Lab, Research Center “Principe Felipe”, C/ Eduardo Primo Yufera 3, 46012 Valencia, Spain; 4 Department of Organic Chemistry, University of Valencia, 46100 Burjassot, Spain; 5 Neuronal and Tissue Regeneration Lab, Research Center “Principe Felipe”, C/ Eduardo Primo Yufera 3, 46012 Valencia, Spain; 6 Institute of Experimental Medicine, Department of Neuroregeneration, Czech Academy of Sciences, Prague, Czech Republic; 7 Department of Human Genetics, Faculty of Medical Sciences, University of Kragujevac, Kragujevac, Serbia; 8 Present Address: Eaton Peabody Laboratories, Department of Otolaryngology, Massachusetts Eye and Ear, Boston, MA USA; 9 Present Address: Department of Otolaryngology-Head and Neck Surgery, Harvard Medical School, Boston, MA USA

**Keywords:** Spinal cord injury, stem cells, neurogenesis, axonal growth, GSK3 inhibition

## Abstract

The inhibition of glycogen synthase kinase-3 (GSK-3) can induce neurogenesis, and the associated activation of Wnt/β-catenin signaling via GSK-3 inhibition may represent a means to promote motor function recovery following spinal cord injury (SCI) via increased astrocyte migration, reduced astrocyte apoptosis, and enhanced axonal growth. Herein, we assessed the effects of GSK-3 inhibition *in vitro* on the neurogenesis of ependymal stem/progenitor cells (epSPCs) resident in the mouse spinal cord and of human embryonic stem cell–derived neural progenitors (hESC-NPs) and human-induced pluripotent stem cell–derived neural progenitors (hiPSC-NPs) and *in vivo* on spinal cord tissue regeneration and motor activity after SCI. We report that the treatment of epSPCs and human pluripotent stem cell–derived neural progenitors (hPSC-NPs) with the GSK-3 inhibitor Ro3303544 activates β-catenin signaling and increases the expression of the bIII-tubulin neuronal marker; furthermore, the differentiation of Ro3303544-treated cells prompted an increase in the number of terminally differentiated neurons. Administration of a water-soluble, bioavailable form of this GSK-3 inhibitor (Ro3303544-Cl) in a severe SCI mouse model revealed the increased expression of bIII-tubulin in the injury epicenter. Treatment with Ro3303544-Cl increased survival of mature neuron types from the propriospinal tract (vGlut1, Parv) and raphe tract (5-HT), protein kinase C gamma–positive neurons, and GABAergic interneurons (GAD65/67) above the injury epicenter. Moreover, we observed higher numbers of newly born BrdU/DCX-positive neurons in Ro3303544-Cl–treated animal tissues, a reduced area delimited by astrocyte scar borders, and improved motor function. Based on this study, we believe that treating animals with epSPCs or hPSC-NPs in combination with Ro3303544-Cl deserves further investigation towards the development of a possible therapeutic strategy for SCI.

**Electronic supplementary material:**

The online version of this article (10.1007/s13311-020-00928-0) contains supplementary material, which is available to authorized users.

## Introduction

Spinal cord injury (SCI) can prompt the loss of motor, sensory, and autonomic functions, while the limited levels of endogenous repair lead to poor functional recovery; however, various studies have highlighted the therapeutic potential of various types of stem/progenitor cells [21, 48, 53]. While some approaches employ the transplantation of neural stem/progenitor cells into the injured spinal cord, other studies focus on the activation of endogenous regenerative machinery. Strategies include treatment with multipotent ependymal stem/progenitor cells (epSPCs) that reside in the central canal of the spinal cord, which can be combined with pharmacology as an alternative strategy [3, 62].

Various signaling mechanisms, including Wnt, notch, sonic hedgehog, growth and neurotrophic factors, bone morphogenetic proteins, neurotransmitters, transcription factors, and epigenetic modulators, are involved in the regulation of adult neurogenesis [23]. In our previous study, we demonstrated the modulation of the inhibitory consequences of astrogliosis following the transplantation of human embryonic stem cell–derived neural progenitors (hESC-NPs), possibly through the secretion of protective factors and the activation of notch and JAK/STAT signaling, as a means to favor neurogenesis [21]. Of note, glycogen synthase kinase-3 (GSK-3) inhibition has been linked to an increase in the neurogenesis in mouse NPs *in vitro* and adult mouse brains [40, 45]. Additionally, the activation of Wnt/β-catenin signaling via GSK-3 inhibition may represent a means to promote motor function recovery from SCI via increased astrocyte migration, reduced astrocyte apoptosis, and enhanced axonal growth [15, 18, 58, 59].

In our new study, we aimed to assess the effects of GSK-3 inhibition on the neurogenesis of the epSPCs resident in the mouse spinal cord, hESC-NPs, and human-induced pluripotent stem cell–derived neural progenitors (hiPSC-NPs), which we refer to collectively as human pluripotent stem cell–derived neural progenitors (hPSC-NPs) throughout the study. We chose the Ro3303544 GSK-3 inhibitor due to its low toxicity, high potency, and potentially beneficial effects on SCI regeneration by the stimulation of astrocyte migration [58]. However, to assess the neurogenic effects of this inhibitor *in vitro* and to facilitate *in vivo* evaluations, we synthesized a more soluble hydrochloride salt form of Ro3303544 (Ro3303544-Cl). We then tested a racemic mixture of both Ro3303544-Cl enantiomers in a spinal cord transection animal model of SCI. Overall, *in vivo* treatment with Ro3303544-Cl increased the expression of neuronal markers at the injury epicenter and adjacent regions and increased neurogenesis, thus partly explaining the improvement in locomotor recovery observed in Ro3303544-Cl–treated animals after severe SCI.

## Results

### GSK-3 Inhibition by Ro3303544 Stimulates *In Vitro* Neurogenesis

epSPCs, multipotent neural precursors that reside in the spinal cord, can proliferate, migrate, and differentiate in response to injury [47, 48]. The potentiation and activation of this endogenous machinery could contribute to the rescue of neurological function after SCI [47]. We tested Ro3303544, a potent activator of astrocyte migration [58], to discern whether this GSK-3 inhibitor could also induce neurogenesis in epSPC cultured *in vitro*.

**Fig. 1:**
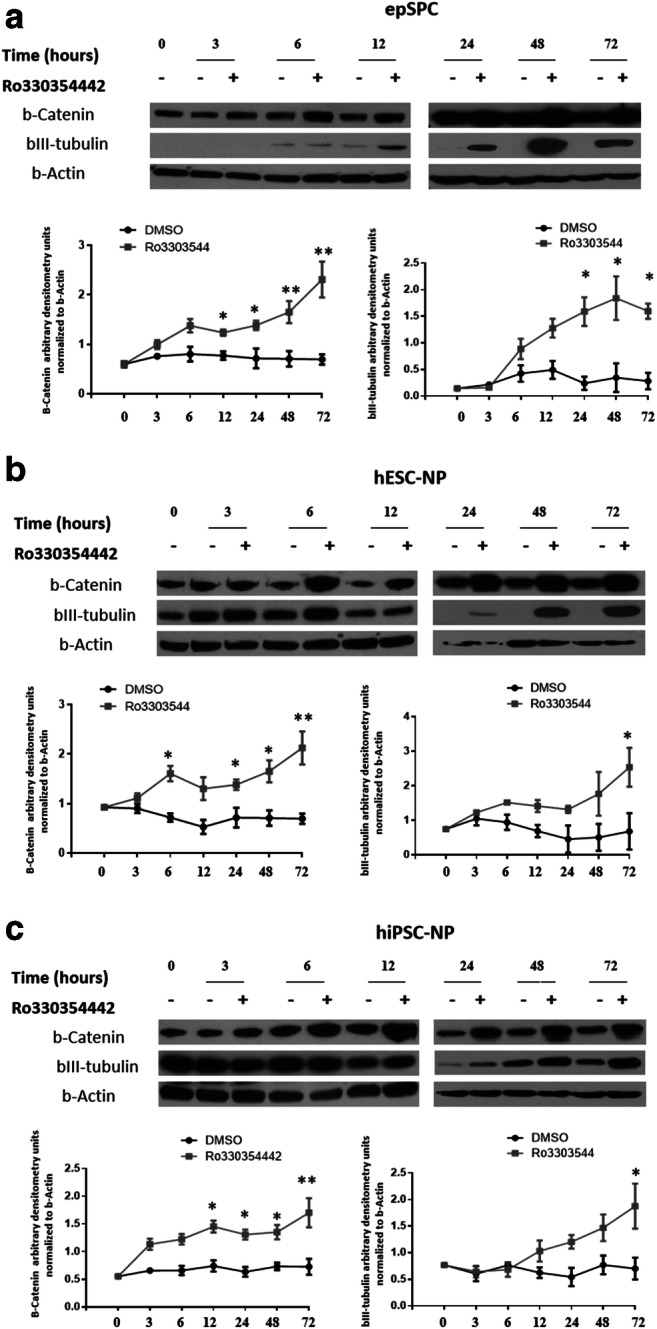
Treatment with Ro3303544 inhibits GSK-3 and increases the expression of the bIII-tubulin neurogenesis marker. Expression of β-catenin, as a control for GSK-3 inhibition, and the neuronal marker bIII-tubulin in epSPCs (**A**), hESC-NPs (**B**), and hiPSC-NPs (**C**) treated with Ro3303544 and compared to DMSO-treated vehicle control cells. β-Actin was used as a loading control, and the results represent data from three independent experiments. Data represent the mean ± standard deviation. Significant statistical comparisons: *p < 0.05; **p < 0.01. Abbreviations: DMSO, Dimethyl sulfoxide; epSPCs, ependymal stem/progenitor cells; hESC-NPs, human embryonic stem cell–derived neural progenitors and hiPSC-NPs, human- induced pluripotent stem cell–derived neural progenitors

We treated epSPCs with vehicle (dimethyl sulfoxide (DMSO) as control) or Ro3303544 (1 μM) for 0 h, 3 h, 6 h, 12 h, 24 h, 48 h, and 72 h to determine the exact timing of GSK-3 inhibition, as shown by the increased expression of β-catenin (Fig. 1A). GSK-3 inhibition resulted in the activation of β-catenin expression after 3 h and became statistically significant after 12 h (*p* < 0.05). Levels of the bIII-tubulin neurogenesis marker significantly increased when compared with those of control cells after 24 h (*p* < 0.05) (Fig. 1A).

**Fig. 2:**
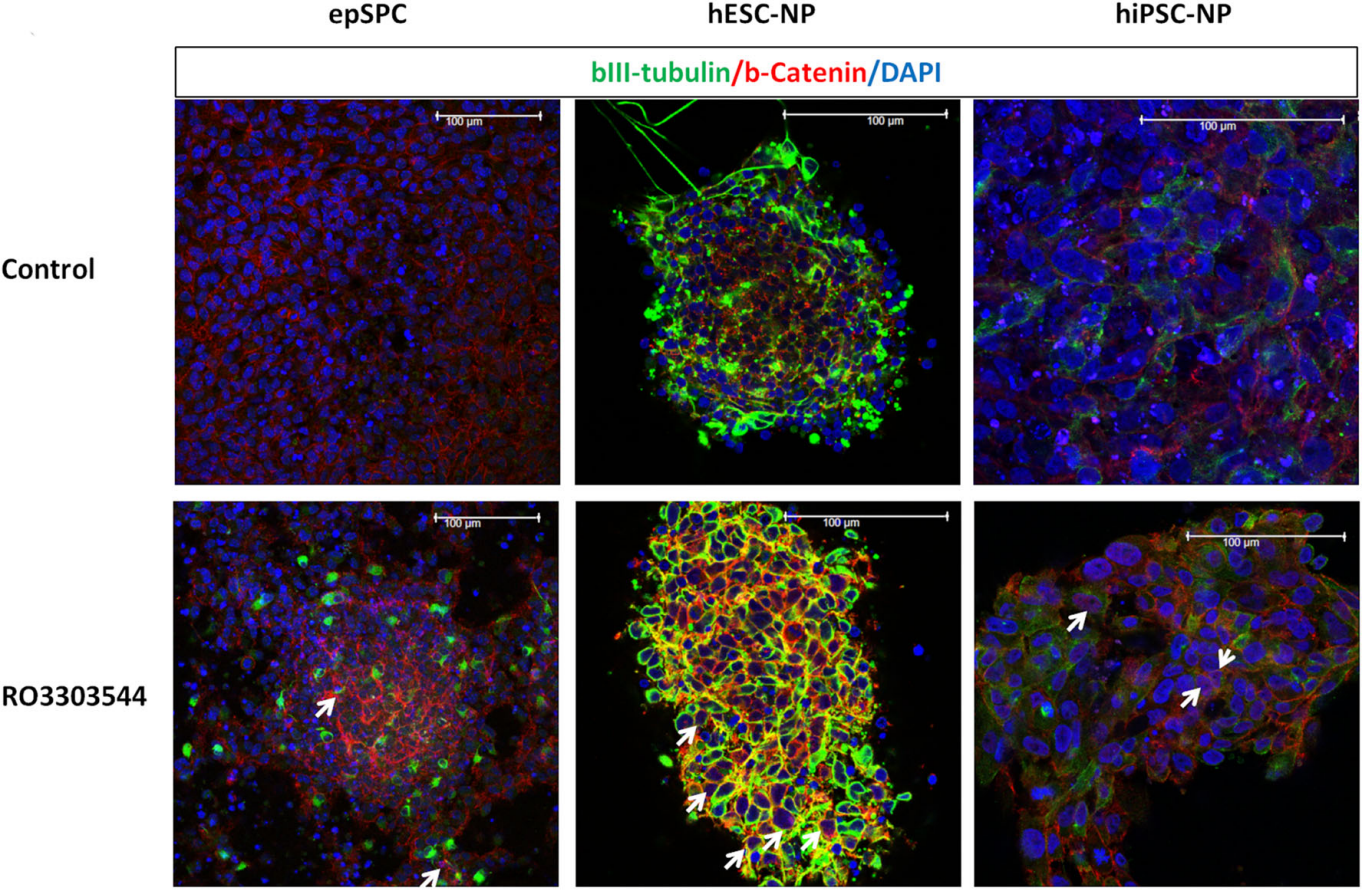
Representative immunocytochemical analysis of epSPCs and hPSC-NPs for β-catenin (red), bIII-tubulin (green), and DAPI (blue). Arrows mark nuclear β-catenin. Scale bars = 100 μm.  Abbreviations: epSPCs, ependymal stem/progenitor cells; hESC-NPs, human embryonic stem cell–derived neural progenitors and hiPSC-NPs, humaninduced pluripotent stem cell–derived neural progenitors; DAPI, 4′,6-diamidino-2-phenylindole

The transplantation of exogenous stem cells, such as hPSC-NPs, represents an alternative means of replacing lost neurons and oligodendrocytes or providing a microenvironment that favors axonal growth [42, 64]. Recently, we developed a simple, animal-free protocol for the neural conversion of both hPSC in adherent culture conditions [41]. A simple medium formula induces the direct conversion of > 98% of hESCs and hiPSCs after 27 days into expandable, transplantable, and functional neural progenitors with neural rosette characteristics (hPSC-NPs) [41]. We examined the effect of Ro3303544 on hPSC-NPs as candidates for cell transplantation, observing a significant increase in the expression level of β-catenin in hESC-NPs after 6 h and in hiPSC-NPs after 12 h when compared to control cells (*p* < 0.05). The inhibition of GSK-3 also resulted in a significant increase in levels of the bIII-tubulin neurogenesis marker in hPSC-NPs after 72 h (*p* < 0.05) (Fig. 1B, C). As expected, we observed by immunocytochemistry that Ro3303544-mediated GSK-3 inhibition caused nuclear and perinuclear accumulation of β-catenin in epSPCs (cultured for 2 weeks) and hPSC-NPs (after differentiation for 27 days) (Fig. 2).

### Ro3303544 Promotes Differentiation Towards Mature Neurons

**Fig. 3:**
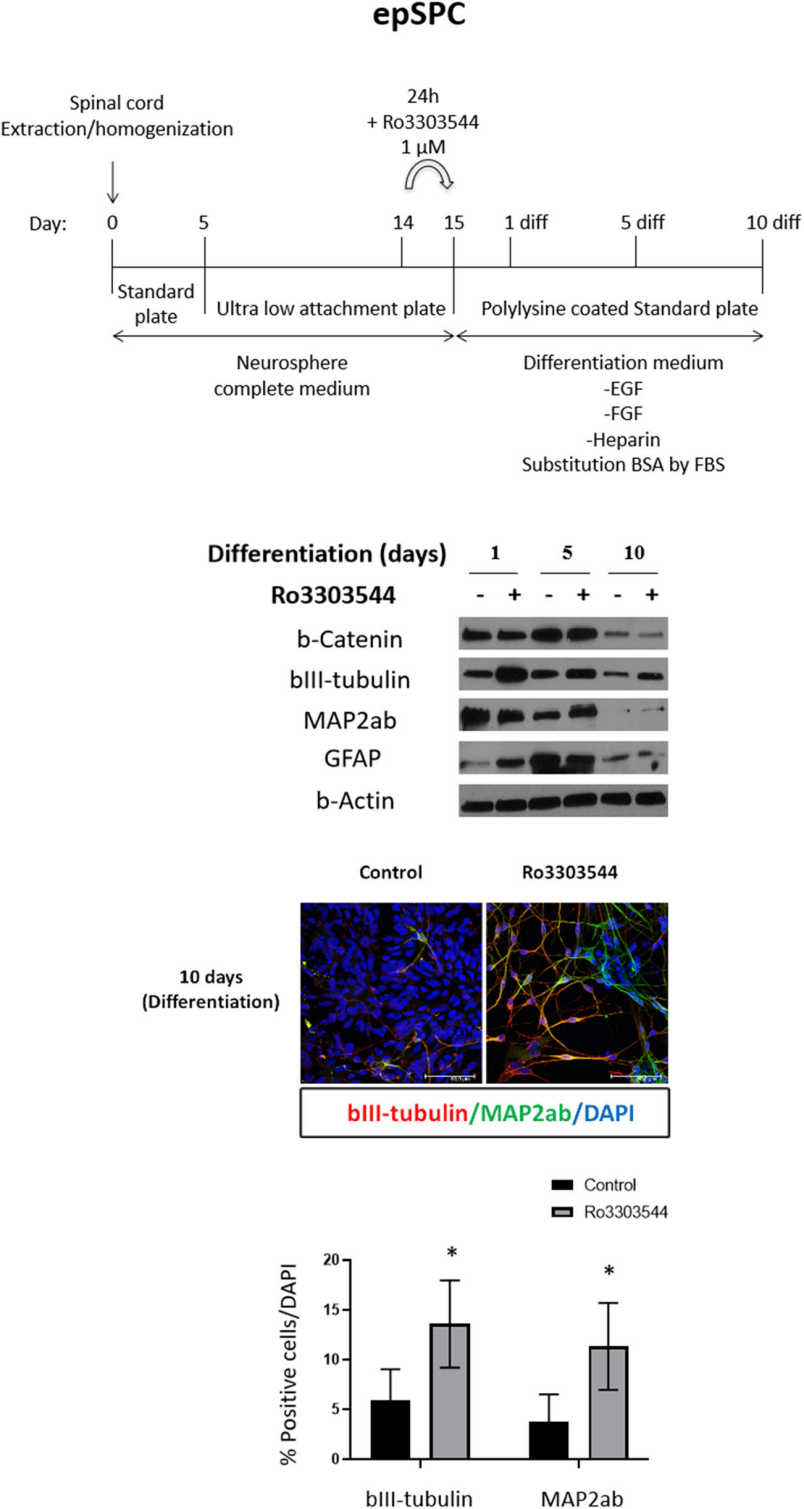
Effects of Ro3303544 during the differentiation of mouse epSPCs. Upper panels show a schematic representation of epSPC culture, drug treatment, and the final differentiation process towards mature neurons. The drug treatment of epSPCs was performed with Ro3303544 or DMSO for 24 h. Expression of β-catenin, the bIII-tubulin early neuronal marker, the MAP2ab postmitotic terminally differentiated neuron marker, and the GFAP astrocytic marker were analyzed by Western blotting after 1 day, 5 days, and 10 days of differentiation. β-Actin was used as a loading control. A representative image of epSPCs differentiated for 10 days in each experimental condition demonstrates the expression of bIII-tubulin and MAP2ab. Immunopositive cells for each neuronal marker were quantified and are represented in the graph. Data represent the mean ± standard deviation. Significant statistical comparisons: *p < 0.05. Abbreviations: diff, differentiation; EGF, Epidermal Growth Factor; FGF, basic Fibroblast Growth Factor; BSA, Bovine Serum Albumin; FBS, Fetal Bovine Serum; epSPCs, ependymal stem/progenitor cells; GFAP,Glial Fibrillary Acidic Protein; MAP2ab, Microtubule Associated Protein 2ab; DAPI, 4′,6-diamidino-2-phenylindole

To determine the effects of Ro3303544 on neuronal differentiation, we treated epSPCs and hPSC-NPs and then induced differentiation into mature neuronal lineage cells. We cultured epSPCs from the spinal cord for 14 days [60] and then treated epSPCs for 1 day with DMSO or Ro3303544 (1 μM). We maintained epSPCs under neuronal differentiation conditions and harvested them after 1 day, 5 days, and 10 days of differentiation (Fig. 3, upper panel). We quantified protein expression by Western blotting and densitometry analysis using the ImageJ Gel Analysis tool (data not shown). After 1 day of neuronal differentiation, we observed a robust increase in the expression of bIII-tubulin (*p* < 0.001) in Ro3303544-treated cells (Fig. 3); however, the expression of bIII-tubulin decreased progressively during differentiation. The increase in MAP2ab expression became significant after 5 days of differentiation in the epSPCs treated with Ro3303544 (*p* < 0.05) compared to control cells.

We also quantified additional markers for other neural lineage cells, including GFAP (astrocytes). After an initial significant increase in GFAP following Ro3303544 treatment compared to control cells (at day 1) (*p* < 0.05), the expression of GFAP decreased at the final differentiation endpoint (Fig. 3).

Representative images after 10 days of epSPC differentiation demonstrated the expression of the bIII-tubulin and MAP2ab neuronal markers in Ro3303544-treated epSPCs when compared to control cells (Fig. 3). We quantified the percentage of positive cells for both marker and found a significant increase in Ro3303544-treated cells compared to control cells after 10 days of differentiation (*p* < 0.05) (Fig. 3).

**Fig. 4:**
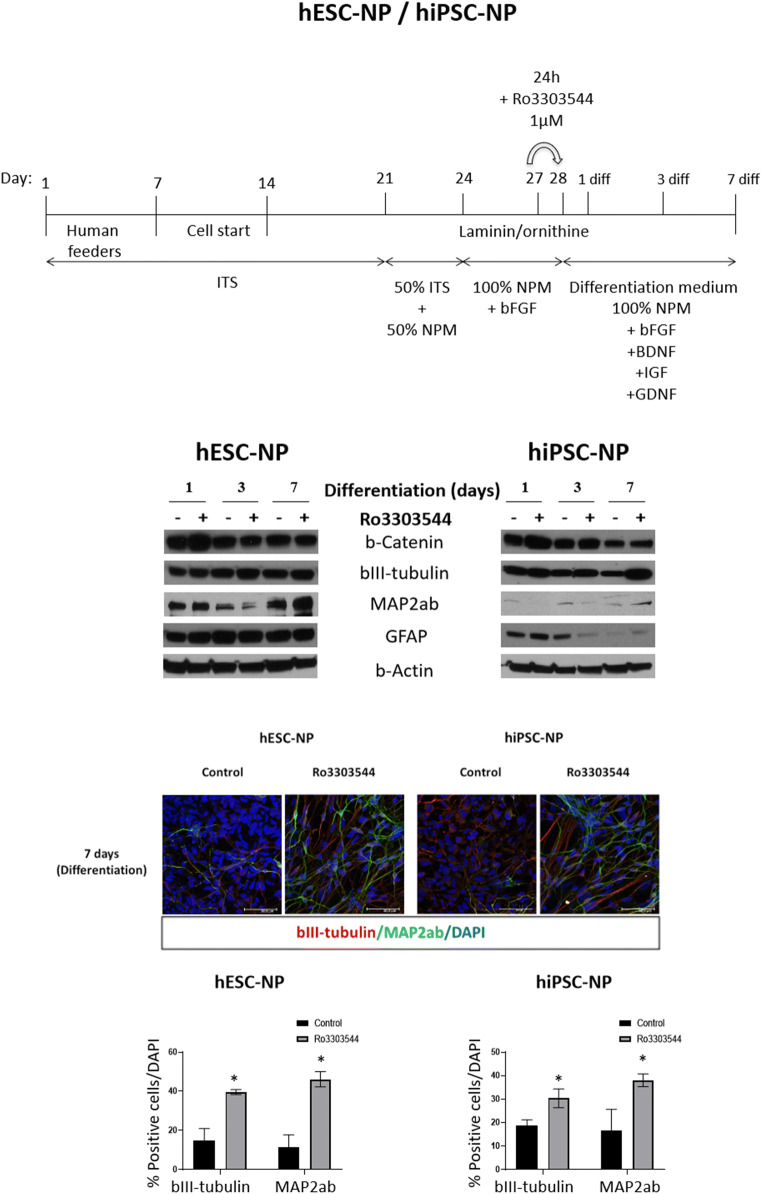
Effects of Ro3303544 treatment during the differentiation of hPSC-NPs. Upper panels show a schematic representation of hESC-NPs and hiPSC-NPs in culture, during drug treatment and during differentiation towards mature neurons. Drug treatment of hESC-NPs and hiPSC-NPs was performed with Ro3303544 or DMSO for 24 h. Expression of β-catenin, the bIII-tubulin early neuronal marker, the MAP2ab postmitotic terminally differentiated neuron marker, and the GFAP astrocytic marker were analyzed by Western blotting after 1 day, 3 days, and 7 days of the differentiation process. β-Actin was used as a loading control. A representative image of hPSC-NPs differentiated for 10 days in each experimental condition shows the expression of neuronal markers bIII-tubulin and MAP2ab. Immunopositive cells for each neuronal marker were quantified and are represented in the graph. Data represent the mean ± standard deviation. Significant statistical comparisons, *p < 0.05. Abbreviations: diff, differentiation; FGF, basic Fibroblast Growth Factor; BDNF, brain-derived neurotrophic factor; GDNF, glial cell line–-derived neurotrophic factor; IGF, insulin-like growth factor; ITS, a mixture of recombinant human Insulin, human Transferrin, and Sodium Selenite; NPM, neural proliferation medium; hESC-NPs, human embryonic stem cell–derived neural progenitors and hiPSC-NPs, human- induced pluripotent stem cell–derived neural progenitors; GFAP, Glial Fibrillary Acidic Protein; MAP2ab, Microtubule Associated Protein 2ab; DAPI, 4′,6-diamidino-2-phenylindole

We differentiated hPSCs into NPs for 27 days and then treated resultant hPSC-NPs with DMSO or Ro3303544 for 1 day (Fig. 4, upper panel). We then maintained hPSC-NPs in neural differentiation medium containing recombinant factors that direct differentiation towards postmitotic neurons, which included brain-derived neurotrophic factor (BDNF), glial cell line–derived neurotrophic factor (GDNF), and insulin-like growth factor (IGF). We discovered that the expression of β-catenin (*p* < 0.05) and bIII-tubulin (*p* < 0.05) in hPSC-NPs significantly increased in comparison to control cells 1 day after the initiation of differentiation (Fig. 4). We also observed a significant increase in the expression of the MAP2ab mature neuron marker after 7 days of differentiation in hPSC-NPs (*p* < 0.05) (Fig. 4). Furthermore, we observed a significant induction in GFAP expression after 1 day of differentiation in both hPSC-NPs (*p* < 0.05) (Fig. 4).

Representative images after 7 days of hPSC-NP differentiation demonstrated the induced expression of the neuronal markers bIII-tubulin and MAP2ab in Ro3303544-treated cells in comparison to control cells. We quantified the percentage of cells positive for both markers in control and Ro3303544-treated cells (Fig. 4). We observed a significantly higher percentage of positive cells for bIII-tubulin and MAP2ab in differentiated cells derived from Ro3303544-treated hPSC-NPs (*p* < 0.05) (Fig. 4).

Overall, these results indicate that Ro3303544 favors the conversion of early murine and human neuronal progenitors into mature postmitotic neurons.

### Racemic Mixture of Ro3303544 Enhances Neurogenesis *In Vitro*

According to the patent, Ro3303544 is a racemic mixture of enantiomers dissolved in DMSO. In many cases, the two enantiomers of chiral molecules have different effects and activity [22, 70]. For the preparation of both enantiomeric forms of Ro3303544, we introduced the asymmetric part of the final structure by the direct addition of the corresponding chiral reagent, thereby maintaining chirality (Supplementary Fig. 1).

**Fig. 5:**
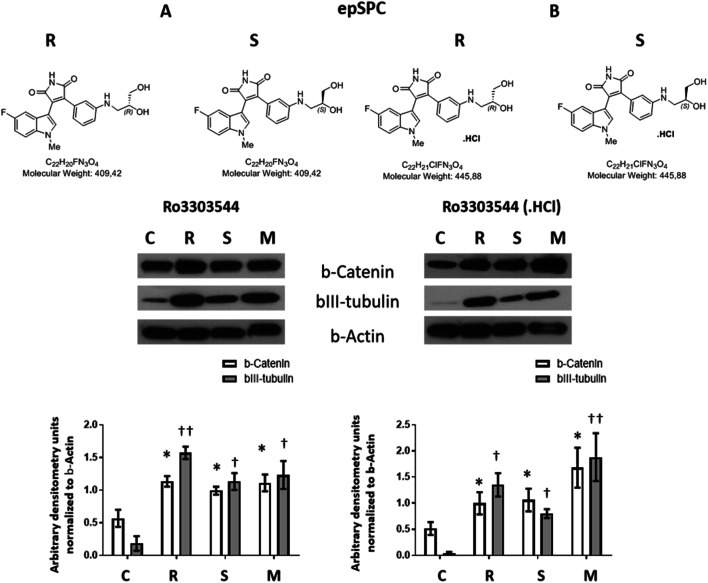
Evaluation of the activity of Ro3303544 (**A**) and Ro3303544-Cl (**B**) enantiomers and a racemic mixture in the expression of β-catenin and bIII-tubulin in epSPCs. Enantiomers (R and S) of Ro3303544 and Ro3303544-Cl were synthesized separately, and equal quantities of R and S mixed in a racemic mixture (M) were compared to vehicle control (C). The compounds (1 μM) were evaluated for the induction of bIII-tubulin expression in epSPCs by Western blotting (panels) and quantified by densitometry (graphs). β-Actin was used as a loading control. Data represent the mean ± standard deviation from three independent experiments. Significant statistical comparisons:, *p < 0.05; †*p* < 0.05; ††*p* < 0.01. Abbreviations: epSPCs, ependymal stem/progenitor cells

To assess the activity of each Ro3303544 enantiomer (R or S), we synthesized both enantiomers in independent reactions (Supplementary Fig. 1). We treated epSPCs with each enantiomer of Ro3303544 (1 μM) separately or with a racemic mixture (1:1) (M) for 1 day. All Ro3303544 preparations significantly enhanced the expression of β-catenin (*p* < 0.05) and bIII-tubulin (S and M; *p* < 0.05) (R; *p* < 0.01) in comparison to vehicle-treated control cells (Fig. 5A).

To avoid the adverse effects associated with DMSO treatment [56], we synthesized efficient, highly bioavailable, nontoxic, water-soluble Ro3303544 derivatives for *in vivo* administration (hydrochloride salt of Ro3303544 (Ro3303544-Cl); Supplementary Fig. 1). Both enantiomers of Ro3303544-Cl (R and S) enhanced the expression of β-catenin and bIII-tubulin (R and S; *p* < 0.05) in epSPCs when compared to the vehicle-treated (0.9% saline) control cells (C). Ro3303544-Cl racemic mixture (M) treatment led to a significantly higher expression of β-catenin (*p* < 0.05) and bIII-tubulin (*p* < 0.01) compared to control (Fig. 5B).

Given these results, we employed a racemic mixture of Ro3303544-Cl for our *in vivo* experiments in a mouse model of SCI (complete spinal cord transection).

### Effects of Ro3303544 on Neurogenesis in an Animal Model of Spinal Cord Injury

**Fig. 6:**
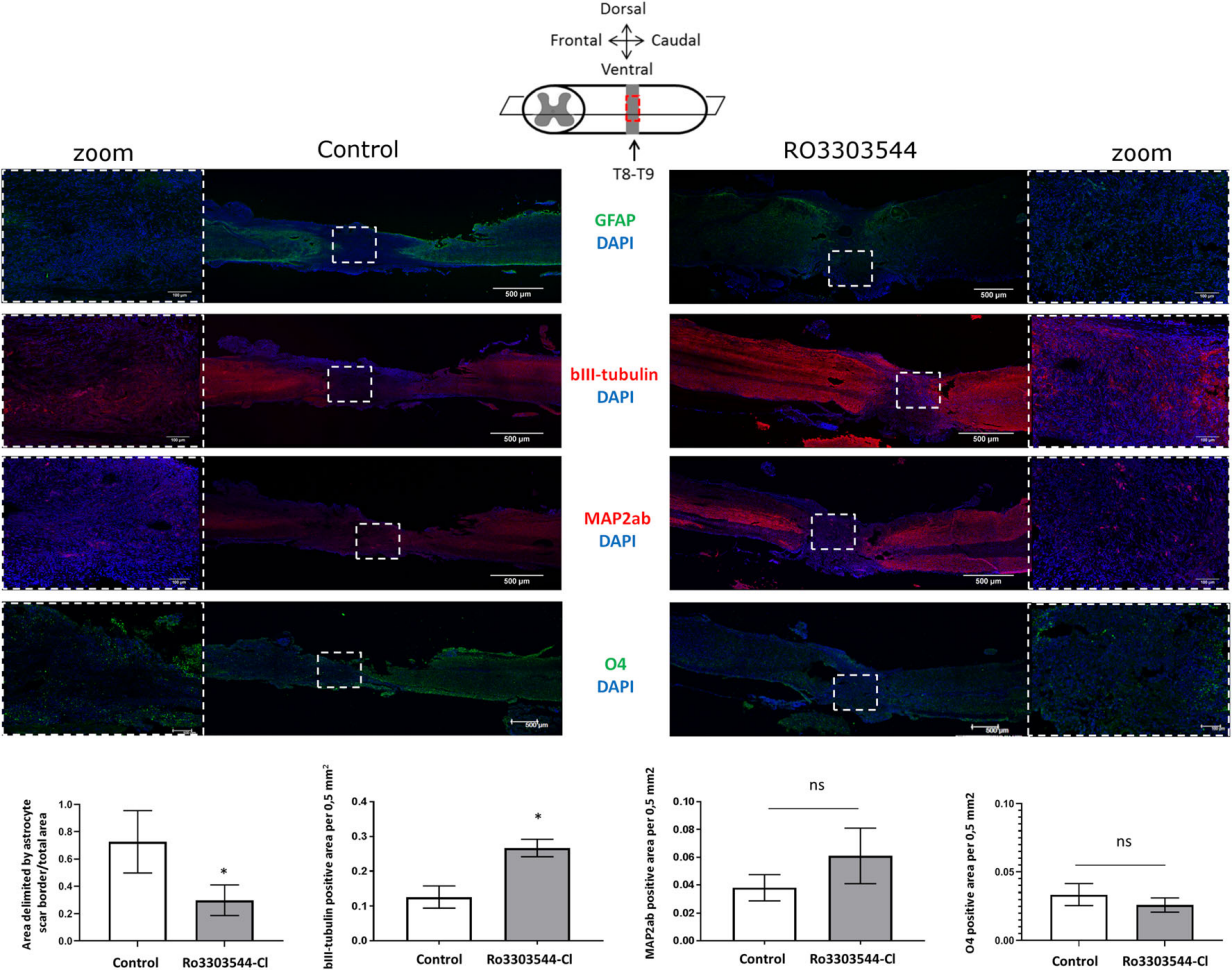
*In vivo* effects of Ro3303544-Cl in the injury epicenter after complete transection of the spinal cord. 0.9% saline (vehicle control) and a racemic mixture of Ro3303544-Cl (500 μM in 0.9% saline) were administered 5 days after SCI. Consecutive longitudinal and central sections (horizontal plane) (10 μm) were used for GFAP, bIII-tubulin, MAP2ab, and O4 antibody staining. Immunohistochemical evaluation of the expression of the astrocyte lineage marker GFAP signal identifies the epicenter of injury. Detection of the bIII-tubulin early neuronal marker and the MAP2ab mature neuron marker was performed in injured spinal cord tissues from animals sacrificed 60 days post injury. The dotted window indicates the position of the magnified image. The scale bar corresponds to 500 μm in a longitudinal section and 100 μm in zoom. GFAP was used to quantify the area delimited by the astrocyte scar border, and the positive area for bIII-tubulin, MAP2ab, and O4 was quantified by ImageJ software and is represented in the graphics. Data represent the mean ± standard deviation. Significant statistical comparisons (*n* = 6 per group), *p < 0.05; *ns* = not significant. Abbreviations: T8, thoracic vertebra 8; T9, thoracic vertebra 9; GFAP, Glial Fibrillary Acidic Protein; MAP2ab, Microtubule Associated Protein 2ab; O4, Oligodendrocyte Marker O4; DAPI, 4′,6-diamidino-2-phenylindole

We intraperitoneally administered Ro3303544-Cl in mice for 5 days after the complete transection of the spinal cord [58]. We evaluated the *in vivo* effects of Ro3303544-Cl at 60 days after complete transection of the spinal cord. We performed immunohistochemical analysis within the injured epicenter for markers of neuronal lineage (bIII-tubulin and MAP2ab for neurons, GFAP for astrocytes, and O4 for mature oligodendrocytes) at 60 days post injury and quantified the staining by ImageJ analysis (Fig. 6). GFAP immunodetection allows the identification of the injury epicenter area, and we quantified the area delimited by astrocyte scar borders in serial longitudinal sections. We observed a significant reduction in the GFAP-delimited area in animals treated with a racemic mixture (1:1) of Ro3303544-Cl (*p* < 0.05) (Fig. 6). Furthermore, animals treated with Ro3303544-Cl displayed significantly higher levels of immunostaining for the early neuronal marker bIII-tubulin within the injury epicenter (*p* < 0.05) (Fig. 6). We also observed a slight but nonsignificant increase in the expression of the MAP2ab, a terminally differentiated neuronal marker, in the injured epicenter (Fig. 6). However, we failed to find any differences in the expression of the O4 oligodendrocyte marker between Ro3303544-Cl–treated and control animals (Fig. 6).

**Fig. 7:**
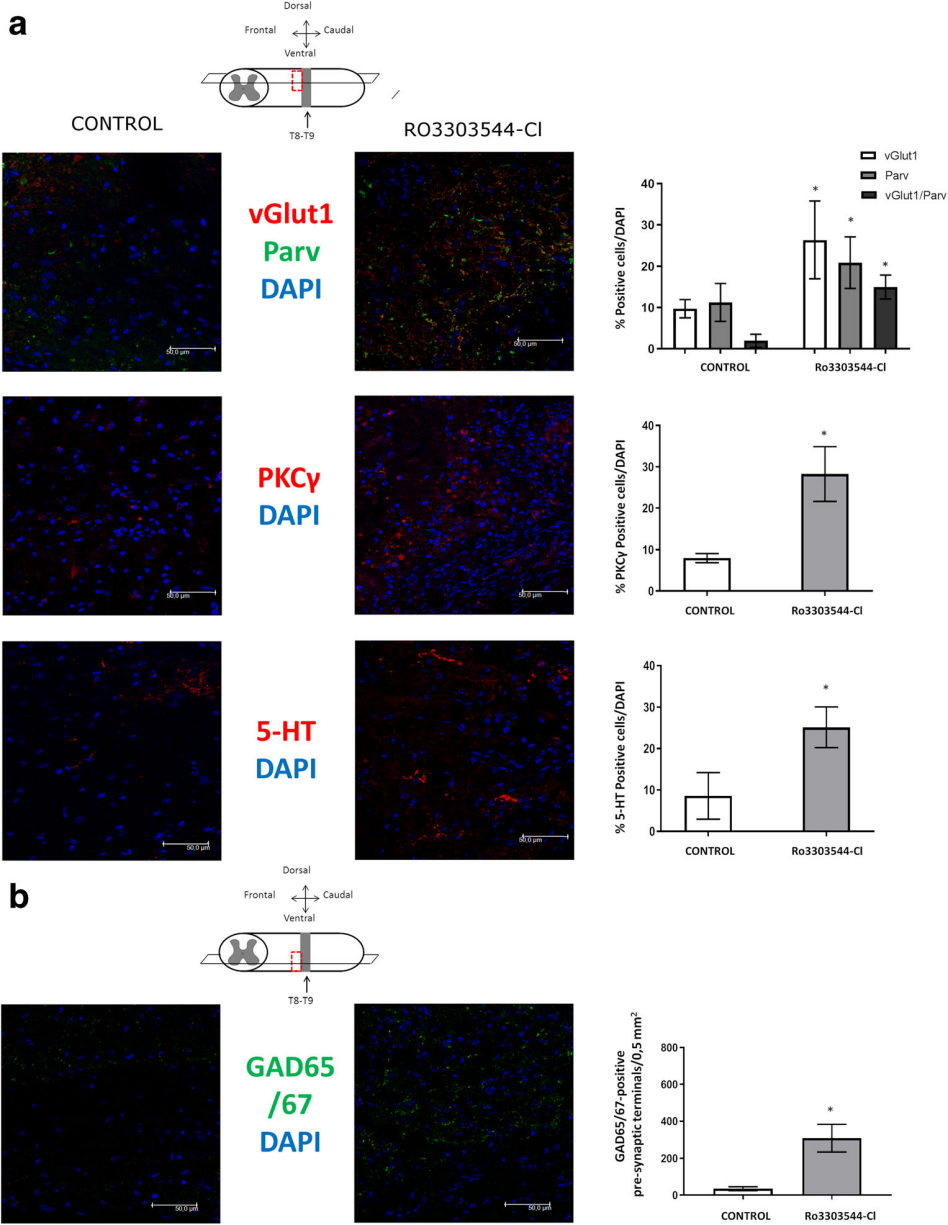
*In vivo* outcomes of Ro3303544-Cl treatment in adjacent regions rostral to injury epicenter. (**A**) Evaluation of protein expression of vGlut1 and Parv to mark propriospinal neurons, PKCγ to mark corticospinal tract (CST), and 5-HT to mark raphe tract by immunohistochemistry in dorsal sections (horizontal plane). The percentage of positive cells/total cells for vGlut1, Parv, PKCγ, and 5-HT is normalized to DAPI in the presence or absence of the drug. Each marker was quantified and is represented in the graphs from dorsal sections. (**B**) Evaluation of GABAergic interneurons used protein detection of GAD65/67 in ventral sections. Positive presynaptic terminals/0.5 mm^2^ were quantified and are represented in the graphic. Data represent the mean ± standard deviation. Significant statistical comparisons (*n* = 6 per group), *p < 0.05. The scale bar in the images corresponds to 50 μm. Abbreviations: T8, thoracic vertebra 8; T9, thoracic vertebra 9; vGlut1, Vesicular glutamate transporter 1; Parv, Parvalbumin; PKC-γ, Protein Kinase C Gamma; 5-HT, 5-Hydroxytryptamine (serotonin); GAD65/67, Glutamic acid decarboxylase 65/67; DAPI, 4′,6-diamidino-2-phenylindole

We also evaluated tissue preservation in the regions adjacent to the injury epicenter. We explored dorsal sections (horizontal plane) of tissue above (cervical) the injury site to analyze the survival of propriospinal, corticospinal, and raphe tracts after Ro3303544-Cl treatment. We enumerated the number of cells expressing markers for propriospinal neurons, such as vesicular glutamate transporter (vGlut1), for myelinated afferent fibers and corticospinal inputs [75], and parvalbumin (Parv), the most commonly used marker of proprioceptive (muscle) afferents [25, 51]. The co-localization of both proteins together marks proprioceptive afferents [80]. We observed a significant increase in the number of cells positive for vGlut1 and Parv in the region above the injury of animals treated with Ro3303544-Cl, indicating an elevated number of sensory fibers [51] (*p* < 0.05) (Fig. 7A). We also detected the significantly higher expression of both the gamma isotype of protein kinase C (PKCγ), a marker of corticospinal tract [20, 67] and interneurons of the inner part of lamina II of the dorsal horn [50] (Fig. 7A, Supplementary Fig. 2), and serotonin (5-hydroxytryptamine or 5-HT), a marker of raphe neurons, above the injury site (*p* < 0.05) [27] (Fig. 7A).

We also evaluated the expression of glutamic acid decarboxylase 65 and 67 (GAD65/67), a marker for GABAergic interneurons that mediates presynaptic inhibition [32]. We discovered significantly higher levels of GAD65/67 expression in ventral sections in the region above the injury in animals treated with Ro3303544-Cl (*p* < 0.05) (Fig. 7B) that could restore GABA content after nerve injury.

**Fig. 8:**
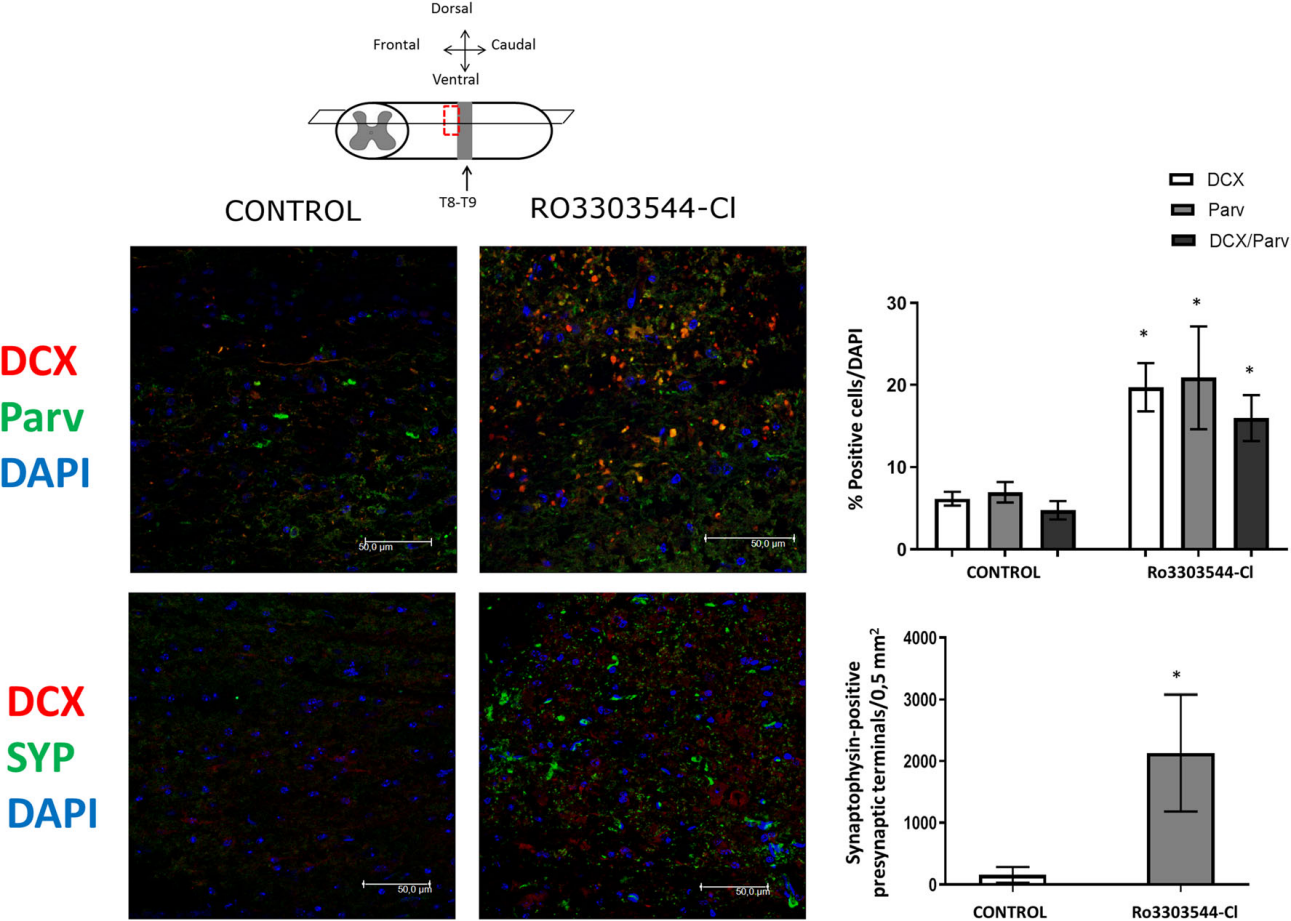
Quantification of newborn neurons *in vivo* induced by Ro3303544-Cl treatment in the adjacent region rostral to injury epicenter in dorsal sections (horizontal plane). Immunolocalization of newborn neurons labeled by DCX (red), inhibitory interneurons labeled by Parv (green), and dual-labeled neurons (yellow). The percentage of positive cells/total cells quantified by DAPI for DCX/Parv-positive cells is represented in the upper graph. Immunolocalization for DCX (red) and SYP (green) is shown, and SYP-positive presynaptic terminals/0.5 mm^2^ were quantified and are represented in the lower graph. Data represent the mean ± standard deviation. Significant statistical comparisons (*n* = 6 per group), *p < 0.05. The scale bar in the images corresponds to 50μm. Abbreviations: T8, thoracic vertebra 8; T9, thoracic vertebra 9; DCX, Doublecortin; Parv, Parvalbumin; SYP, Synaptophysin; DAPI, 4′,6-diamidino-2-phenylindole

We evaluated not only the putative role of Ro3303544-Cl in the maintenance of tracts and interneurons after injury but also its role in neurogenesis. For that purpose, we employed antibodies against doublecortin (DCX), a widely used marker for newborn neurons/adult neurogenesis in mice and humans [17, 33, 35]. We observed a significantly higher number of newborn neurons marked by DCX alone or DCX and Parv in regions adjacent to the injury epicenter in animals treated with Ro3303544-Cl when compared to control animals (*p* < 0.05). These results indicate that DCX-positive cells are, in part, inhibitory proprioceptive Parv-positive interneurons (Fig. 8). To investigate the functionally of newly generated neurons, we explored the expression of the synaptic marker synaptophysin (SYP). We found higher SYP expression (above the injury, *p* < 0.05) in Ro3303544-Cl–treated animals when compared to control animals. Even though we found SYP expression near newly generated neurons labeled by DCX, we failed to observe any double labeling (Fig. 8).

**Fig. 9:**
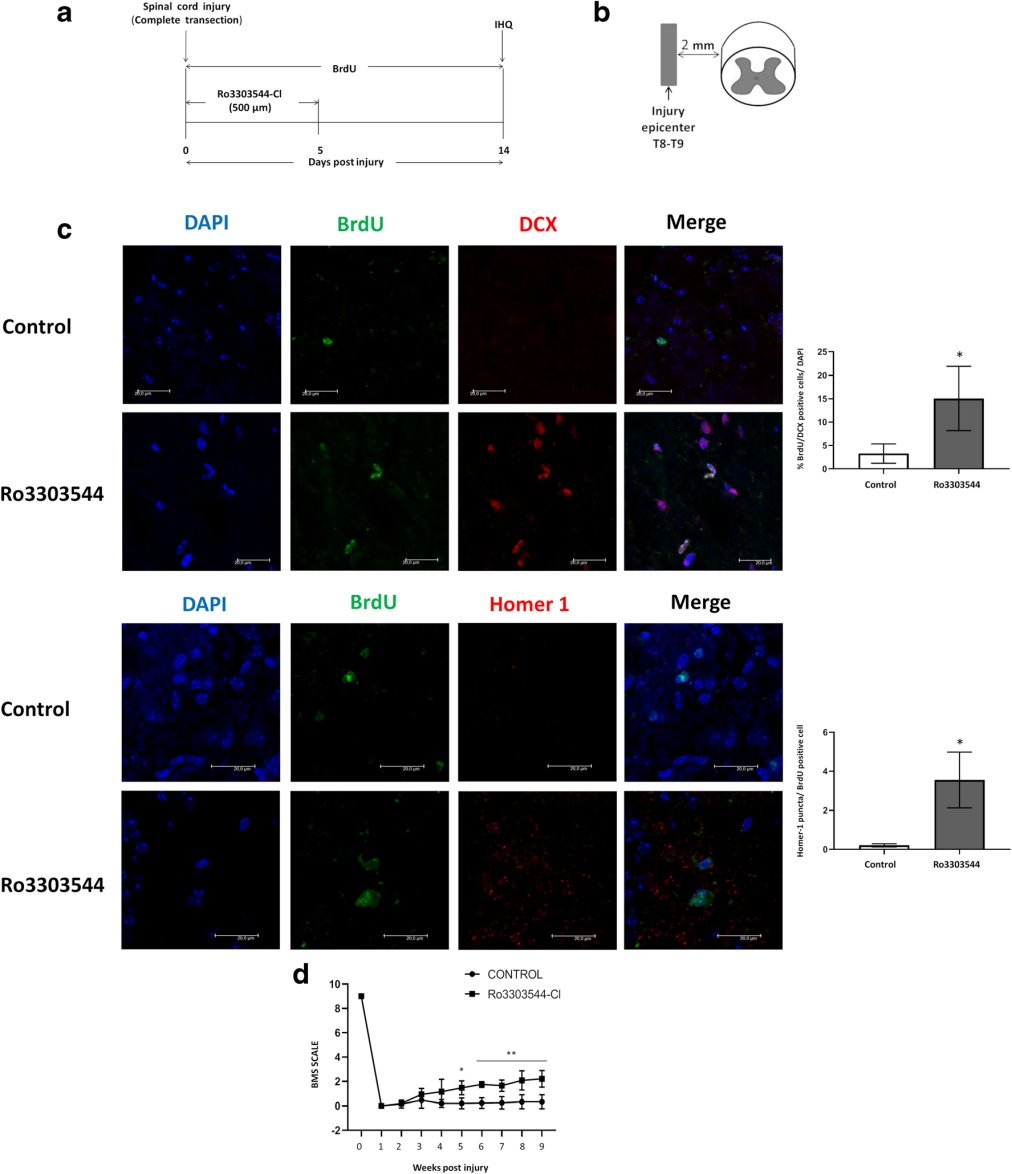
BrdU-traced newly generated neurons and integration in neuronal circuits in the injured spinal cord. (**A**) Schematic representation of the experimental timeline *in vivo*. For studies of neurogenesis, BrdU injection occurred from 0 to 14 days and Ro3303544-Cl treatment from 0 to 5 days. (**B**) Schematic diagram showing the position for transverse sections (2 mm) caudal to the lesion site. (**C**) Quantification of BrdU/DCX-positive cells (labeled by arrows) detected in transverse sections (10 μm) below the injury epicenter in control and Ro3303544-Cl–treated animals (*n* = 6 per group). Quantification of Homer1 postsynaptic terminals per BrdU-positive cell indicates the synapsis of the newly generated neurons detected in transverse sections (10 μm) caudal to the epicenter in the vehicle control (0.9% saline) and Ro3303544-Cl–treated animals (*n* = 6 per group). Significant statistical comparisons, *p < 0.05. Scale bar = 20 mm. (**D**) Locomotor evaluation of injured mice was performed once per week for 60 days post injury and quantified using the Basso Mouse Scale (BMS) rating score. Data represent the mean ± standard deviation (0.9 % saline and Ro3303544-Cl) (*n* = 6 per group). Significant statistical comparisons:, *p < 0.05; **p <0.01. Abbreviations: T8, thoracic vertebra 8; T9, thoracic vertebra 9; IHC, Immunohistochemistry; BrdU = 5-bromo-2′-deoxyuridine; DCX, Doublecortin; Homer1, Homer Scaffold Protein 1; DAPI, 4′,6-diamidino-2-phenylindole

To demonstrate the existence of immature postmitotic neurons, we employed immunohistochemistry with double staining for 5-bromo-2′-deoxyuridine (BrdU) (to label dividing cells) and DCX in transverse sections caudal to the epicenter. We labeled dividing cells in tissues from vehicle-treated and Ro3303544-Cl–treated animals with BrdU for 14 days after SCI [58] (Fig. 9A, B). Animals treated with Ro3303544-Cl (first 5 days after SCI) demonstrated a significant increase in the number of BrdU/DCX double-positive cells in comparison to control animals (*p*<0.05) (Fig. 9C). To demonstrate that newly generated neurons participate in functional recovery after SCI, we explored their integration in neuronal circuits by synapsis formation. We employed double-staining immunohistochemistry using transverse sections caudal to the epicenter for BrdU and Homer1, a postsynaptic density scaffolding protein. We enumerated the number of Homer1 postsynaptic terminals per BrdU-positive cell in control or Ro3303544-Cl–treated animals; this analysis revealed a significant increase in the number of boutons for each newborn neuron that receives synapses from other spinal neurons (*p*<0.05) (Fig. 9C).

Finally, we video recorded mice once per week for locomotion recovery analysis. We analyzed locomotor activity in an open field using the Basso Mouse Scale (BMS) rating scale, finding a significant increase in the locomotion of hind limbs in animals treated with Ro3303544-Cl at 5 weeks (*p* < 0.05) and 2 months after severe SCI in comparison to nontreated animals (*p* < 0.01) (Fig. 9D).

## Discussion

In this study, we investigated whether the treatment of neural precursors with a potent GSK-3 inhibitor (Ro3303544) could induce neurogenesis and/or the expression of neuronal markers *in vitro* and *in vivo* (with a soluble form of this inhibitor, Ro3303544-Cl) in mice with complete transection of the spinal cord. We observed increased expression of the bIII-tubulin neuronal marker in Ro3303544-treated neuronal precursors in culture, while further differentiation of Ro3303544-treated cells prompted higher levels of markers of terminally differentiated neurons in comparison to vehicle-treated control cells. After complete transection of the spinal cord, animals treated with a clinically relevant derivative of Ro3303544 (Ro3303544-Cl) presented with higher expression of bIII-tubulin within the injury epicenter. Also, we discovered tissue preservation after Ro3303544-Cl treatment and the increased expression of protein markers for the propriospinal tract, raphe tract, and corticospinal tract or PKCγ-positive interneurons and GABAergic interneurons in regions adjacent to the injury epicenter. We also observed higher adult neurogenesis after SCI in animals treated with Ro3303544-Cl as well as improved recovery of locomotion. Overall, we believe that Ro3303544-Cl treatment may contribute towards the development of an efficient therapeutic approach to SCI.

The inhibition of GSK-3 signaling represents a promising means to modulate astrogliosis and induce neurogenesis [24, 72, 73] and may exert synergetic effects in SCI recovery when combined with neural precursor cell therapy [58]. Overall, the administration of GSK-3 inhibitors may facilitate the development of an effective treatment for injuries, including spinal cord trauma [18]. The contribution of ependymal cells to spinal cord repair has been shown in mammalian and nonmammalian animals [37, 57] and even mammals during early development [39]. The neurogenic potential of spinal cord stem cells is restricted to resident neural precursors (epSPCs) during adulthood. This resident stem cell population, located in the areas surrounding the central canal [7, 44], becomes activated by traumatic SCI. epSPCs can self-renew and differentiate into astrocytes and oligodendrocytes [7, 44]. While research has suggested that the glial scar has an inhibitory influence on self-repair and neuroregeneration after SCI, recent studies have established that ependymal cells, inflammatory cells, and astrocytes also possess pro-regenerative properties [4, 7, 63]. Resident epSPCs impair the formation of cysts and restrict secondary damage; moreover, the inhibition of epSPC proliferation heavily compromises the formation of the glial scar after SCI and detrimentally affects neuronal survival [68]. Kim et al. [34] demonstrated that the conditional deletion of GSK-3 increases the proliferation of mouse NPs, while another study indicated that GSK-3 inhibition increases neurogenesis in human NPs [36]. Furthermore, a recent study established that the pharmacological inhibition of GSK-3 induced neurogenesis in the adult rat brain [45].

The use of Ro3303544 in our study is based on a previous report that established a lower toxicity and more potent activity when compared to other GSK inhibitors [58]. Ro3303544 accelerates the migration of reactive astrocytes to sequester inflammatory cells that spare myelinated fibers. Also, the authors of this previous study reported that Ro3303544 promoted neurite outgrowth of embryonic hippocampal neurons *in vitro*, thereby promoting functional recovery after SCI with no noted toxicity [58]. However, published studies have yet to describe the effects of Ro3303544 on neurogenesis in spinal cord-resident epSPCs and hPSC-NPs or after severe SCI.

In this study, we report that Ro3303544 treatment causes a rapid and robust inhibition of GSK-3 that is followed by the induction of β-catenin expression and increased neurogenesis *in vitro* in epSPCs and hPSC-NPs. We also observed increased protein expression of neuronal markers, such as the early neuronal marker bIII-tubulin and the mature neuronal marker MAP2ab. Ro3303544 also enhances differentiation towards mature neurons *in vitro*, as evidenced by increased MAP2ab expression.

In many cases, two enantiomers of chiral molecules interact differently with biological targets, leading to differences in their pharmacology, toxicology, pharmacokinetics, and metabolism [22, 70]. We tested enantiomeric forms of Ro3303544 dissolved in DMSO, which has shown relative cytotoxicity [56], and the more bioavailable, water-soluble hydrochloride salt form of Ro3303544 (Ro3303544-Cl). We included an analysis of Ro3303544-Cl to facilitate prospective *in vivo* experiments, as this form of the drug allows for easier administration and better biodistribution with fewer side effects related to the solvent. In our studies *in vitro*, we show that both enantiomers (R and S) of Ro3303544 or Ro3303544-Cl and their racemic mixture exert a comparable GSK-3 inhibition activity.

Tissue samples from injured animals treated with Ro3303544-Cl presented a reduction in the area delimited by the astrocyte scar borders, in line with previous results with Ro3303544 [58], but little or no effect on the oligodendrocyte population. We explored the effects of Ro3303544-Cl in the maintenance of long tracts after SCI. PKCγ co-localizes with corticospinal tract fibers [2] and has been used to investigate corticospinal tract sprouting and plasticity in SCI models [9, 11]. The increment of PKCγ in tissues from animals treated with Ro3303544-Cl may suggest a putative role in the protection of corticospinal neurons after SCI above the injury [69]. However, PKCγ is also present in interneurons of the inner part of lamina II of the dorsal horn and has been implicated in injury-induced allodynia [50]. We observed higher expression in both the dorsal corticospinal tract and dorsal horn, as shown in transverse sections above the injury epicenter. A subset of PKCγ excitatory neurons is the target for Parv in interneurons [55]. Increased Parv expression may influence cell survival [71], as it prevents excitotoxic cell death [43]. The synaptic plasticity in spinal cord is likely to involve contributions from spared proprioceptive afferents, descending motor axons, interneurons, and motor neurons and can result in modest improvements in motor function [74]. An increase in the number of PKCγ excitatory neurons, together with a decrease in inhibitory transmission stimuli mediated by Parv, is critical to the development of mechanical allodynia [55]. Thus, the administration of Ro3303544-Cl triggered the expression of both PKCγ and Parv, which may not increase neuropathic pain. We also discovered a significant increase in GAD65/67, a marker of inhibitory GABAergic neurons, in animals treated with Ro3303544-Cl that may contribute to reestablishing the normal GABAergic circuitry [12, 32] or the modulation of spasticity [30]. Studies of long descending propriospinal tracts in the spinal cord [25] have demonstrated that terminals expressing vGlut1 were myelinated afferent fibers and/or corticospinal inputs [19, 75]. In contrast, those expressing both were classified as proprioceptive afferents [14, 80]. We observed a higher presence of both proteins alone or co-localizing in the same cell, suggesting that Ro3303544-Cl may favor the presence of proprioceptive sensory fibers in adjacent dorsal regions above the injury epicenter.

After SCI, the depletion in 5-HT that marks serotonergic neurons from the raphe nuclei of the brainstem [16] takes place [27]. The disruption of descending serotonergic projections is a major limiting factor that prevents motor function recovery [29]. A recent report has established that after spinal transection in the adult rat, serotonergic pharmacotherapy combined with exercise showed improve motor recovery due to dendritic plasticity [26]. Locomotor activity can be regained in animal models after spinal transection by application of drugs that activate the neuromodulatory 5-HT, as reviewed elsewhere [49]. Treatment with GSK-3b inhibitors in animals with thoracic spinal cord transection significantly induces descending corticospinal and serotonergic axon regeneration and promotes locomotor functional recovery after SCI [18]. The results presented here indicate that Ro3303544-Cl can increase or preserve raphe spinal fibers above the injury epicenter and may contribute to the observed locomotion improvement.

We studied not only the preservation of tracts but also the possible effect of Ro3303544-Cl on neurogenesis. We established a significant increase in DCX-positive cells treated with Ro3303544-Cl and also discovered that a subpopulation of the newly generated neurons marked by DCX also expressed Parv, which may contribute to the reduction of neuropathic pain [55]. The formation of new synapses after denervation caused by SCI may play a role in the recovery of motor function [38]. The higher expression of the SYP presynaptic marker expression in adjacent regions rostral to injury indicates preservation or new synapse formation by Ro3303544-Cl treatment; however, we failed to observe double labeling with DCX. This finding may be because DCX is an early marker of newborn neurons that migrate to different sites before their neuronal maturation and the formation of synaptic connections [77]. However, we did detect the higher expression of SYP in those animals treated with Ro3303544-Cl in close vicinity to newly generated DCX-positive neurons. The higher number of newly formed neurons (BrdU/DCX-positive cells) observed in Ro3303544-Cl–treated mice could functionally contribute to improved motor performance. However, BrdU appears to label only a small fraction of DCX-positive cells, which leaves open a possibility that DCX-positive cells are preserved rather than newly generated. Some of the new neurons might eventually become integrated into the local circuitry and function as interneurons, as evidenced by the significantly higher number of BrdU-positive cells expressing the Homer1 postsynaptic marker that could contribute to enhanced neurological outcomes and spinal cord recovery. Studies have shown that the local activation of the circuitry within the lumbar spinal segments, known as central pattern generators (CPGs), can produce stepping-like movements in the hind limbs after anatomically complete and incomplete SCI [6, 8]. Some authors consider that the circuitry connecting skin and muscle primary afferents with spinal interneurons and motoneurons is active after complete SCI, where sensory information is transmitted to the animal’s spinal cord [76]. After complete spinal transection, there exists the possibility that intrinsic spinal mechanisms formed by a network of interneurons coupled to increased sensory feedback cooperate to enhance the recovery of locomotion as a result of spinal cord neural plasticity, as previously described in cats [52, 65, 66, 76]. Functional recovery from motor and sensory deficits can occur spontaneously [31] or be promoted by different treatments [13], including pharmacological agents [65]. Thus, it remains conceivable that the high levels of neuron markers for the mentioned tracts after Ro3303544-Cl treatment might be associated with locomotor recovery and sensory circuitry improvements as a result of increased spinal cord plasticity.

One significant limitation of the study is the lack of functional sensory evaluations to test allodynia and/or sensory recovery; further studies will be required to assess the effects of treatment on nociceptive and/or mechanical stimuli.

## Conclusions

Overall, our results suggest that the treatment of spinal cord-resident mouse ependymal stem cells (epSPCs) and human neural progenitors derived from pluripotent stem cells (hESC-NP and hiPSC-NP) with a GSK-3 inhibitor (Ro3303544) increases neurogenesis, as reflected by the increased expression of β-catenin and neuronal markers (bIII-tubulin [early] and MAP2ab [mature]). Administration of a soluble, bioavailable form of this GSK-3 inhibitor (Ro3303544-Cl) reduced the area delimited by astrocyte scar borders, enhanced neurogenesis, and exerted a putative neuroprotective effect *in vivo* at 2 months after injury. The primary outcome of Ro3303544-Cl treatment is the improvement of functional locomotor recovery in animals with severe SCI. Future evaluations will study Ro3303544-Cl treatment in combination with stem cell therapy to examine if this approach constitutes a feasible strategy for SCI regeneration.

## Material and Methods

### Synthesis of Ro3303544, Ro3303544-Cl, and Enantiomeric Forms

Ro3303544 was developed and kindly provided by Roche (Department of Genetics and Genomics, Roche, Palo Alto, CA). The initial characterization of Ro3303544 has been previously reported [1]. The synthesis of Ro3303544 results in a racemic mixture [28, 54]. *In vitro* experiments using mouse epSPC and hPSC-NPs compared the effect of the aforementioned racemic mixture of Ro3303544 (1 μM) dissolved in DMSO with a control solution that included an equivalent concentration of DMSO (0.1%).

To obtain a nontoxic and more water-soluble drug for *in vivo* evaluation, we synthesized the enantiomeric forms and a racemic mixture of the hydrochloride salt of Ro3303544 (Ro3303544-Cl). We determined the activity of each enantiomer separately and a mixture of equal quantities of each enantiomer for Ro3303544 and Ro3303544-Cl in epSPC.

However, to carry out the preparation of all the forms of this compound in the laboratory, we had to modify its synthetic route due to the inaccessibility of specific reactants, as well as the low yields observed in intermediate steps. The preparation of intermediate 5 started with the methylation of commercially available fluorinated indole 1 with CH_3_I in the presence of NaH in dimethylformamide (DMF) (Supplementary Fig. 1). The resulting *N*-methylated indole was then treated with oxalyl chloride in Et_2_O to generate *N*-methyl indole-3-glyoxylyl chloride 2 at a 75% yield over two steps, which was used without further purification. At this point in the synthesis, we attempted to find an alternative route to that previously described in the literature. Following a new strategy [78], compound 2 was treated with sodium methoxide in MeOH, generating intermediate 3 at a 60% global yield from 1. The ^1^H-NMR signals are in accordance with those described in the literature for this product [79]. For the next step, commercially available 3-nitrophenylacetic acid was treated with SOCl_2_ and NH_4_OH to obtain 2-(3-nitrophenyl)acetamide 4 at a yield of 60%. The ^1^H-NMR signals are in accordance with those described in the literature for this product [46]. Subsequent condensation of ester 3 with acetamide 4 using *t*-BuOK in tetrahydrofuran (THF) generated the key intermediate 5 at a yield of 50% (Supplementary Fig. 1A). With intermediate 5 on hand, the nitro group was reduced via hydrogenation catalyzed palladium on carbon (Pd/C) in acetic acid to generate compound 6 (Supplementary Fig. 1B). Reductive amination of intermediate 6 was then carried out by adding to a mixture of (*R*) or (*S*)-2,2-dimethyldioxolane-4-carboxaldehyde 9 in the presence of Na(OAc)_3_BH, affording (*R*)-7a and (*S*)-7b. Chemical de-protection of the diol moiety in (*R*)-7a and (*S*)-7b in acidic media afforded the final compounds (*R*)-8a and (*S*)-8b as separate enantiomers. A comparison of the activity of different enantiomeric forms, (R)-enantiomer, and (S)-enantiomer, and a racemic mixture (M) of Ro3303544 and Ro3303544-Cl was performed by Western blotting analysis. The racemic mixture of Ro3303544-Cl was used for *in vivo* experiments (500 μM).

### Isolation, Culture, and Ro3303544 Treatment of Mouse epSPCs

epSPCs were obtained from the spinal cord of healthy mice, as previously described [47, 61]. Once the overlying meninges and blood vessels were removed, the dissected tissue was cut into 1 mm^3^ pieces and homogenized mechanically. epSPCs were cultured as neurospheres in complete medium, as described elsewhere [47]. Briefly, neurospheres were cultured in growth medium in low-attachment plates (DMEM/F12 supplemented with 100 U/ml penicillin, 100 μg/ml streptomycin, 2 mM l-glutamine, 5 mM HEPES buffer, 0.125% NaHCO_3_, 0.6% glucose, 0.025 mg/ml insulin, 80 μg/ml apotransferrin, 16 nM progesterone, 60 μM putrescine, 24 nM sodium selenite, 4 μg/ml bovine serum albumin (BSA), 0.7 U/ml heparin, 20 ng/ml epidermal growth factor, and 20 ng/ml basic fibroblast growth factor (bFGF). Neurospheres were isolated and cultured for 2 weeks. Neurospheres were then treated with vehicle (DMSO) or Ro3303544 (in DMSO) (1 μM) for 0 h, 3 h, 6 h, 9 h, 12 h, 24 h, 48 h, and 72 h. epSPCs were also used to determine the activity of each enantiomer and a racemic mixture of Ro3303544 (in DMSO) and Ro3303544-Cl (in 0.9% saline).

### Neural Differentiation of Ro3303544-Treated epSPCs

Neurospheres were cultured for 2 weeks and then treated with vehicle (DMSO) or Ro3303544 (in DMSO) (1 μM) for 1 day. Ro3303544 was then removed, and cells were cultured in fresh differentiation medium, described elsewhere [61]. Briefly, heparin, EGF, and FGF were removed from the complete growth medium described above. BSA was replaced by 2% fetal bovine serum (FBS). Under these conditions, epSPCs were forced to differentiate, and after 1 day, 5 days, and 10 days, cells were collected for further analysis by Western blotting.

### Cell Culture of hPSCs

The cell culture of hPSC was performed as previously described [41]. Primary hESC colonies (H9; WiCell, Inc., Madison, WI) or human hiPSC (clone 4) [5] were mechanically dispersed into several small clumps, which were then cultured on fresh commercially available human foreskin fibroblasts (American Type Culture Collection, Manassas, VA) inactivated by irradiation (45 Gy) in hESC medium containing knockout DMEM (Invitrogen, Carlsbad, CA), 100 μM β-mercaptoethanol (Sigma, Ronkonkoma, NY), 1 mM l-glutamine (Invitrogen), 100 mM nonessential amino acids, 20% serum replacement (SR; Invitrogen), 1% penicillin-streptomycin (Invitrogen), and 8 ng/ml bFGF (Invitrogen). The hPSC medium was changed daily. hPSCs were passaged by incubation in 1 mg/ml collagenase IV (animal-free, Invitrogen) for 5 to 8 min at 37 °C or mechanical dissociation and then removed to freshly prepared human foreskin fibroblast layers.

### Differentiation of hPSCs into Neural Progenitor Cells (hPSC-NPs)

Undifferentiated hESCs or hiPSCs were maintained on feeders with hPSC medium for further differentiation to multipotent neural precursors (hESC-NPs and hiPSC-NPs, together named hPSC-NPs). At day 0, hESC medium was changed to ITS medium which contains DMEM/F12, dextran (6%), human insulin 50 μg/ml, holotransferrin 5 ng/ml, sodium selenite (50 ng/ml), Glutamax 1×, taurine (0.5 M), and ascorbic acid (50 μg/ml) and cells were maintained for 7 days with daily medium changes. During this period, neural differentiation begins with the formation of small neural tube-like structures called rosettes, which increase in size and grow in three dimensions. These structures were mechanically separated from surrounding feeder cells using a needle and transferred to plates coated with human-defined matrix CELLstart (Life Technologies, Carlsbad, CA; 1:50 prepared in 6% of dextran) and maintained with ITS medium over the following 7 days. From D14 to D21, cells were disaggregated by Accutase, plated on human laminin/polyornithine-precoated plates, and cultured in ITS medium. Then, hPSC-NPs were maintained in 50% ITS medium and 50% of neural proliferation medium (NPM) for 3 days. NPM consisted of DMEM/F12, XenoFree B-27 supplement (Invitrogen), 25 μg/ml human insulin (Sigma), 6.3 ng/ml progesterone, 10 μg/ml putrescine, 50 ng/ml sodium selenite, and 50 μg/ml human holotransferrin (Sigma). Cells were then cultured in NPM supplemented with 8 ng/ml human recombinant bFGF (Invitrogen) for 3 days. Neural progenitors were then treated with vehicle (DMSO) or Ro3303544 (in DMSO) (1 μM) for 0 h, 3 h, 6 h, 9 h, 12 h, 24 h, 48 h, and 72 h and harvested.

### Neural Differentiation of Ro330354-Treated hPSC-NPs

After 27 days of the above-described differentiation process, hPSC-NPs were treated with Ro3303544 (DMSO) (1 μM) in fresh NPM for 1 day. Ro3303544 was then removed, and hPSC-NPs were forced to differentiate by culture in fresh NPM supplemented with bFGF (8 ng/ml), BDNF (10 ng/ml), IGF-1 (2 ng/ml), and GDNF (10 ng/ml) (PeproTech, Cranbury, NJ). Eventually, cells were collected after 1 day, 3 days, and 7 days for further analysis by Western blotting.

### Western Blotting Analysis

Cells or spinal cord tissue segments (2 cm length; (*n* = 6, per condition) at the injury epicenter were collected and proteins extracted by using lysis buffer (50 mM Tris-HCl, pH 7.5, 150 mM NaCl, 0.02% NaN_3_, 0.1 SDS, 1% NP40, 1 mM EDTA, 2 μg/ml leupeptin, 2 μg/ml aprotinin, 1 mM PMSF, 1× Protease Inhibitor Cocktail) (Roche Diagnostics, Indianapolis, IN). Equal amounts of protein extracts (50 μg) were loaded onto a 10% SDS-polyacrylamide gel and resolved by standard SDS-PAGE. Proteins were electrophoretically transferred onto PVDF membranes. Membranes were blocked with 5% skimmed milk in PBST for 60 min and tested overnight with specific antibodies at the dilution 1:500 of mouse monoclonal anti-bIII-tubulin (MO15013; Neuromics Biotechnology, Edina, MN), mouse monoclonal anti-β-catenin (610153; BD Transduction Laboratories, San Jose, CA), mouse monoclonal anti-MAP2ab (clone AP20 Cat. MAB3418; Millipore, Burlington, MA), mouse monoclonal anti-GFAP (M0761; Dako, Carpinteria, CA), and mouse monoclonal β-actin at dilution 1:5000 (A5441; Sigma Chemical, St. Louis, MO) which was used as a loading control. Subsequently, membranes were incubated with rabbit anti-mouse or rabbit anti-goat horseradish peroxidase–conjugated secondary antibody (1:5000) (Sigma Chemical). Blots were visualized by the ECL detection system (Amersham, Little Chalfont, Buckinghamshire, UK). The results were quantified by densitometry using ImageJ Software.

### Surgical Procedures and Ro3303544-Cl Treatment *In Vivo*

Female adult C57/BL6 (30–60 g) mice were used for surgical procedures and immunohistological studies. Before surgery, animals were subcutaneously premedicated with morphine (1 mg/kg). Mice were then placed in an induction chamber, to which 4% isoflurane was supplied in a continuous oxygen flow of 1 l/min. Once the animals were anesthetized, they were maintained with isoflurane 1 to 2% in a continuous flow of 0.2 to 0.4 l/min of oxygen. The dorsal area between the neck and hind limbs extending ~ 2 cm bilaterally from the spine was shaved and disinfected with serial povidone. Under aseptic conditions, a longitudinal dorsal midline skin incision was made over the spinal column from T5 to T11, and the muscles overlying the vertebral column were reflected, exposing the vertebral columns T7 to T10. The mice were then subjected to a laminectomy at the T8 to T9 thoracic spinal cord segments, and a complete transection of the spinal cord was performed with a microknife (10055-12; FST, Foster City, CA). Before the complete transection, drops of lidocaine were topically administered on the lesion site to reduce local pain. After lesion, the cavity was explored with a glass probe to cut any residual filament and to verify complete transection. The deep and superficial muscle layers were sutured, and the skin was closed with 6/0 sterile suture. Immediately after surgery, the animals were given subcutaneous 0.9% saline, antibiotic Baytril (1 mg/kg/day, s.c.; Bayer, Pittsburgh, PA), and Buprex (0.03 mg/kg, s.c.; Schering-Plough, Kenilworth, NJ) as a painkiller. The animals were maintained on an isothermal pad for better recovery. They were treated with vehicle (0.9% saline) or the racemic mixture of Ro3303544-Cl (0.9% saline) (500 μM) immediately after lesion, twice per day during the following 5 days after SCI. The bladders of all the injured mice were evacuated manually twice per day until bladder function was restored or animals were sacrificed.

Open-field locomotion was evaluated by two independent and blinded assessors, and a consensus score was derived by using the 9-point BMS for locomotion after visualization of a minimum of 3 min of free walking in an open space once a week, starting at 7 days post injury. The Basso Mouse Scale for locomotion detects differences in recovery after spinal cord injury [10]. The animals were inspected for weight loss, dehydration, and discomfort with appropriate veterinary care. Mice whose weight had declined by more than 20% were excluded from experiments (and are not represented in the experimental results). The studies were approved by the CIPF Animal Research Committee and followed the animal care guidelines.

### Immunocytochemical and Immunohistochemical Staining

Cells or tissues were fixed with 4% paraformaldehyde (PFA) at room temperature for 15 min. After the permeabilization of cell membranes with 0.1% Triton X-100, samples were subsequently blocked with PBS containing 10% of FBS. Incubation with primary antibodies was performed overnight (1:200) at 4 °C. Immunohistochemical evaluation was performed 60 days after injury. Mice (*n* = 6, per condition) were transcardially perfused with a 0.9% saline solution followed by 4% PFA in PBS and 2-day incubation time in 30% sucrose before inclusion in Tissue-Tek OCT (Sakura Finetek, Torrance, CA). Tissues were embedded in OCT medium before cutting using a Microm cryostat (HM 505 E). Horizontal or transverse serial sections of spinal cords spanning the injury sites were processed at 10 μm thickness and used for immunohistochemistry. Adjacent sections (two per slide) on the same slide were 50 μm apart. For the horizontal plane, ten serial sections per animal spanning 100 μm of the dorsal–ventral axis of the spinal cord gray matter were used for immunohistochemistry. Cells were quantified in the adjacent region to the epicenter (T8–T9 levels). For the transverse plane, ten serial sections per animal spanning 550 μm of the cervical–caudal axis adjacent to injury epicenter were used for immunohistochemistry. For each section, ten images were captured by confocal microscopy using a confocal microscope (Leica TCS-SP2-AOBS, Wetzlar, Germany), and counts were performed in a blinded manner. Slides of spinal cord sections or cells were processed for immunohistochemistry or immunocytochemical, respectively, using the following primary antibodies when corresponding: rabbit polyclonal anti-GFAP (Z0334, Dako), mouse monoclonal anti-bIII-tubulin (MO15013, Neuromics Biotechnology), mouse monoclonal anti-β-catenin (610153; BD Transduction Laboratories), rabbit polyclonal anti-MAP2ab (ab5622; Chemicon, Temecula, CA), mouse monoclonal anti-O4 (MAB345, Millipore), guinea pig polyclonal anti-vGlut1 (AB5905, Millipore), rabbit anti-Parv (PV27; Swant, Marly, Switzerland), rabbit polyclonal anti-PKCγ (sc-211; Santa Cruz Biotechnology, Dallas, TX), rabbit polyclonal anti-5HT (S5545; Sigma-Aldrich, St. Louis, MO), rabbit polyclonal anti-GAD65/67 (AB1511, Millipore), guinea pig polyclonal anti-DCX (AB2253, Millipore), and mouse monoclonal anti-SYP (sc-17750, Santa Cruz Biotechnology). After wash steps, secondary antibodies (Oregon Green 488 goat anti-mouse IgG, Alexa Fluor 647 mouse anti-rabbit 1:400, or Alexa Fluor 647 goat anti-pig 1:400 (Life Technologies)) in each case were incubated for 1 h at room temperature. Signals were visualized using the fluorescence microscope DM6000 and by confocal microscopy (Leica). The area delimited by the astrocyte scar border was assessed as previously described [47]. Briefly, the quantification of the negative GFAP area (epicenter) was expressed as a percentage of the total area spanning the injury site, equivalent in length (2 mm) for all tested groups.

### BrdU Labeling

Animals were treated with 0.9% saline or Ro3303544-Cl for 5 days after SCI (*n* = 6). The S-phase marker BrdU was used to label cells that underwent proliferation at the time of BrdU injection. BrdU (50 mg/kg body weight) (B5002, Sigma-Aldrich) was intraperitoneally injected daily beginning on the same day of SCI and continuing until 14 days till sacrifice. Animals were anesthetized and transcardially perfused with 0.9% NaCl. Spinal cords then were sequentially cryoprotected in 10%, 20%, and 30% sucrose, and frozen serial transverse sections (10 μm) were prepared on a cryostat. Sections were pretreated with 2 N HCl for 45 min at room temperature, followed by 0.1 M boric acid (pH 8.5) for 10 min at room temperature. Sections were blocked and followed by incubation with mouse anti-BrdU antibody (B8434, Sigma-Aldrich) and rabbit anti-Homer1 (160003; Synaptic Systems, Goettingen, Germany) (1:200; BD Biosciences, San Jose, CA) overnight at 4 °C. For double labeling of BrdU/DCX, sections were first incubated with mouse anti-BrdU antibody overnight at 4 °C. After a series of washes, sections were then incubated with guinea pig polyclonal anti-DCX (AB2253, Millipore) overnight at 4 °C. Finally, sections were rinsed and incubated with secondary antibodies (Oregon Green 488 goat anti-mouse, 647-conjugated goat anti-rabbit, or 647-conjugated goat anti-pig (1:400)) for 1 h at room temperature, rinsed, counterstained with DAPI for visualization of nuclei, and cover-slipped for microscopic evaluation.

### Statistical Analysis

Statistical analysis was performed by using the software GraphPad Prism 5. The Mann–Whitney *U* test was conducted to evaluate the differences between data from control and experimental groups. Nonparametric tests and the Kruskal–Wallis test were performed followed by Dunn’s test for multiple comparisons. A two-way ANOVA with repeated measures followed by a Bonferroni post hoc correction was used in behavioral tests. Values represent the mean ± SD of at least three independent experiments (*p < 0.05, **p < 0.01 for statistically significant differences).

## Electronic Supplementary Material

ESM 1 Synthesis of enantiomeric forms of Ro3303544 and the hydrochloride salt Ro3303544-Cl. Compound 2 was treated with sodium methoxide in MeOH, affording intermediate 3 in 60% global yield from compound 1. Commercially available 3-nitrophenylacetic acid was treated with SOCl_2_ and NH_4_OH to obtain 2- (3-nitrophenyl) acetamide-4. Condensation of ester 3 with acetamide-4 using t-BuOK in tetrahydrofuran (THF) afforded key intermediate 5. The preparation of intermediate 5 by methylation of fluorinated indole-1 with CH3I in the presence of NaH in DMF resulted in an N-methylated indole that was treated with oxalyl chloride in Et_2_O to afford N-methyl indole-3-glyoxylyl chloride-2. Intermediate 5 was reduced via hydrogenation to give compound 6. Reductive amination of intermediate 6 was then carried out by addition to a mixture of (R) or (S)-2, 2-dimethyldioxolane-4-carboxaldehyde-9 in the presence of Na(OAc)_3_BH, affording (R)-7a and (S)-7b. Chemical de-protection of the diol moiety in (R)-7a and (S)-7b in acidic media afforded the final compounds (R)-8a and (S)-8b as separate enantiomers. (PNG 608 kb)

High resolution image (TIF 242 kb)

ESM 2 Precise anatomical localization of PKCγ assessed by immunohistochemistry. Transverse cryosections (10 μm) at 2 mm rostral to the injury epicenter from control and Ro3303544-Cl-treated animals were used to detect the immune-signal of PKCγ (red). DAPI dye was used for routine nuclear staining (blue). DH = Dorsal horn, VH = Ventral horn, CST = corticospinal tract. The scale bar in the images corresponds to 50 or 500 μm. (PNG 4434 kb)

High resolution image (TIF 1505 kb)

ESM 3 (PDF 261 kb)
