# Supplementary figures and images for: Activation of Neurogenesis in Multipotent Stem Cells Cultured In Vitro and in the Spinal Cord Tissue After Severe Injury by Inhibition of Glycogen Synthase Kinase-3
Source: Neurotherapeutics. 2020 Sep 30;18(1):515–33. doi: 10.1007/s13311-020-00928-0 (PMC8116371; doi:10.1007/s13311-020-00928-0)

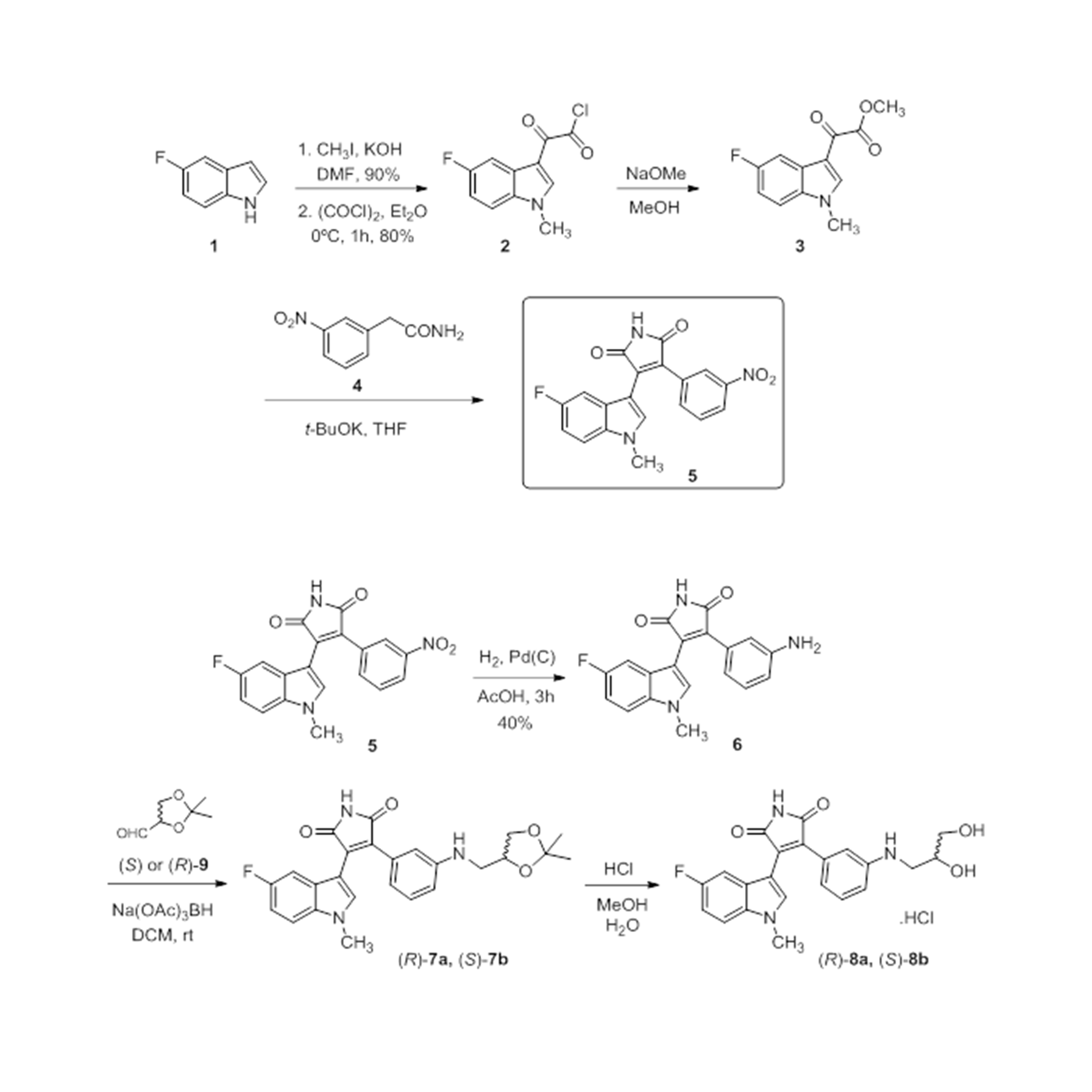

Supplement: Supplementary file 1 — Synthesis of enantiomeric forms of Ro3303544 and the hydrochloride salt Ro3303544-Cl. Compound 2 was treated with sodium methoxide in MeOH, affording intermediate 3 in 60% global yield from compound 1. Commercially available 3-nitrophenylacetic acid was treated with SOCl2 and NH4OH to obtain 2- (3-nitrophenyl) acetamide-4. Condensation of ester 3 with acetamide-4 using t-BuOK in tetrahydrofuran (THF) afforded key intermediate 5. The preparation of intermediate 5 by methylation of fluorinated indole-1 with CH3I in the presence of NaH in DMF resulted in an N-methylated indole that was treated with oxalyl chloride in Et2O to afford N-methyl indole-3-glyoxylyl chloride-2. Intermediate 5 was reduced via hydrogenation to give compound 6. Reductive amination of intermediate 6 was then carried out by addition to a mixture of (R) or (S)-2, 2-dimethyldioxolane-4-carboxaldehyde-9 in the presence of Na(OAc)3BH, affording (R)-7a and (S)-7b. Chemical de-protection of the diol moiety in (R)-7a and (S)-7b in acidic media afforded the final compounds (R)-8a and (S)-8b as separate enantiomers. (PNG 608 kb) [file 13311_2020_928_Fig10_ESM.png]

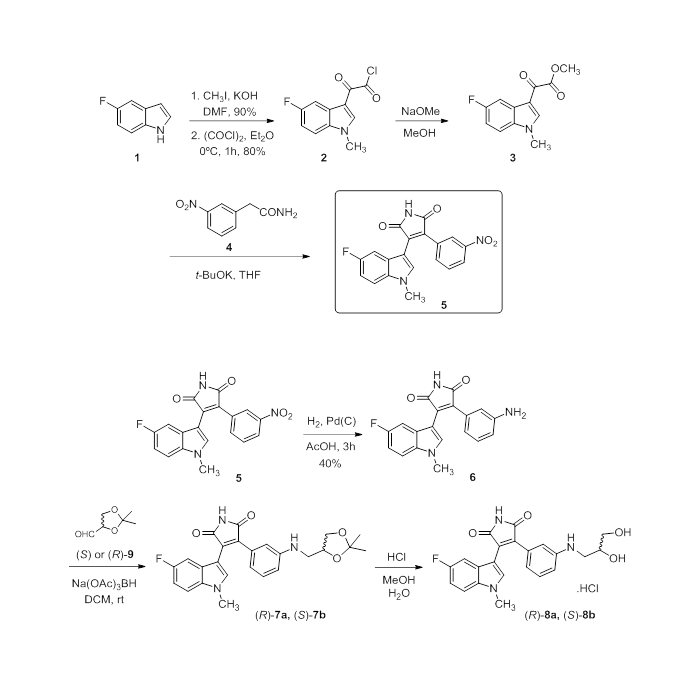

Supplement: Supplementary file 2 — High resolution image (TIF 242 kb) [file 13311_2020_928_MOESM1_ESM.tif]

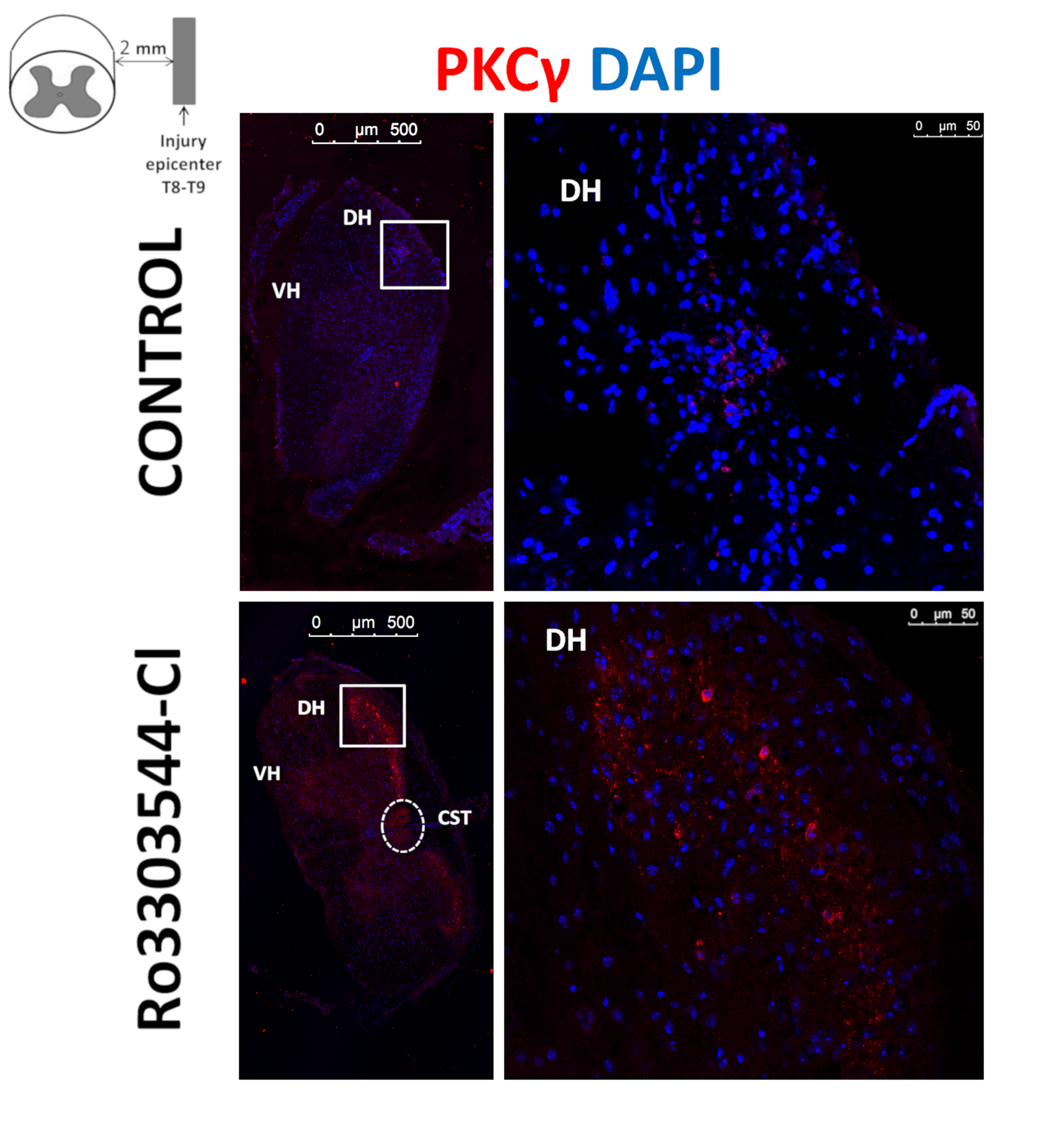

Supplement: Supplementary file 3 — Precise anatomical localization of PKCγ assessed by immunohistochemistry. Transverse cryosections (10 μm) at 2 mm rostral to the injury epicenter from control and Ro3303544-Cl-treated animals were used to detect the immune-signal of PKCγ (red). DAPI dye was used for routine nuclear staining (blue). DH = Dorsal horn, VH = Ventral horn, CST = corticospinal tract. The scale bar in the images corresponds to 50 or 500 μm. (PNG 4434 kb) [file 13311_2020_928_Fig11_ESM.png]

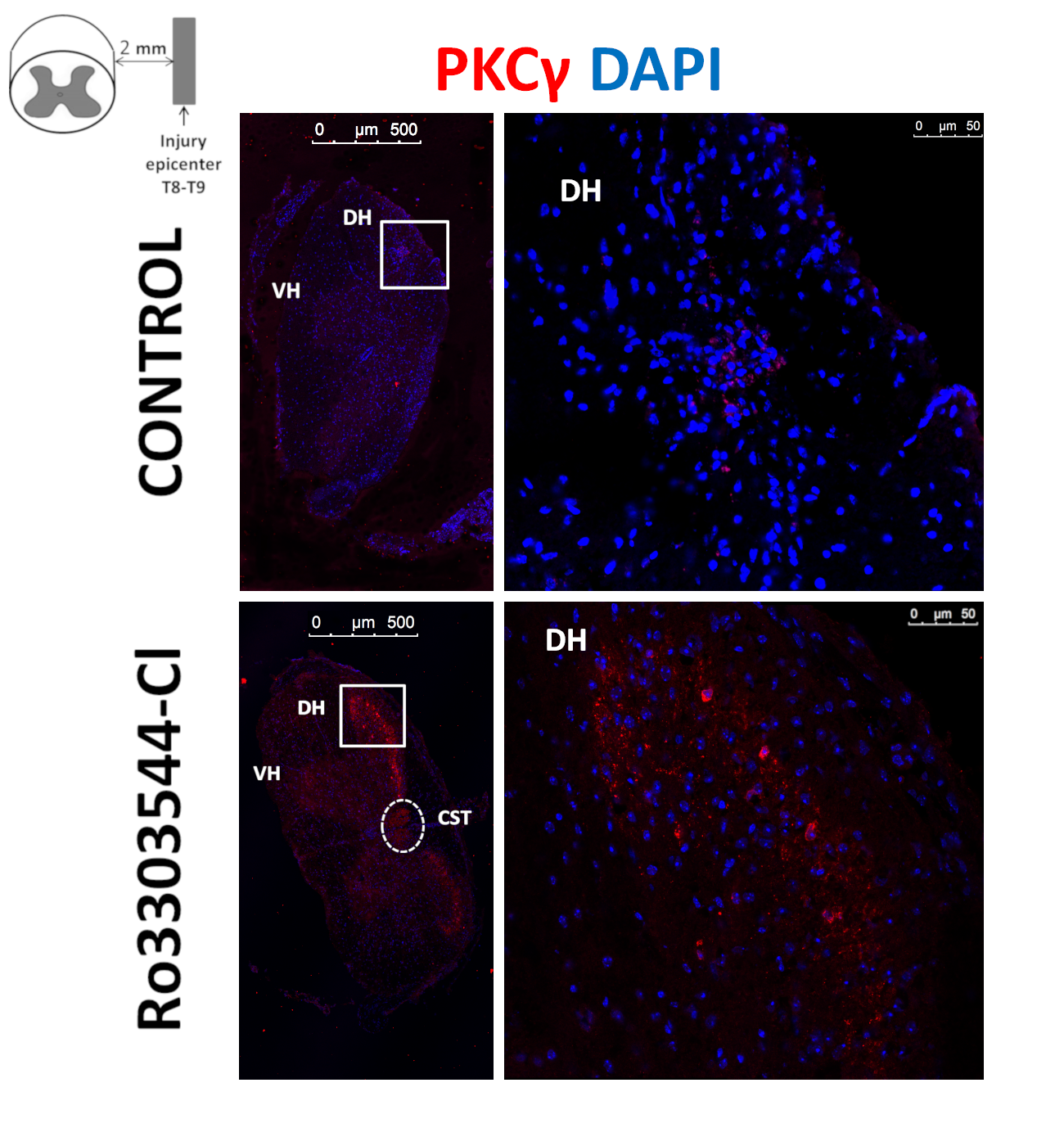

Supplement: Supplementary file 4 — High resolution image (TIF 1505 kb) [file 13311_2020_928_MOESM2_ESM.tif]
